# Prenatal Maternal Psychological Stress (PMPS) and Its Effect on the Maternal and Neonatal Outcome: A Retrospective Cohort Study

**DOI:** 10.3390/healthcare12232431

**Published:** 2024-12-03

**Authors:** Joana Kathleen Aldinger, Harald Abele, Angela Kranz

**Affiliations:** 1 Section of Midwifery Science, Institute of Health Sciences, University of Tübingen, 72076 Tübingen, Germany; harald.abele@med.uni-tuebingen.de (H.A.); angela.kranz@med.uni-tuebingen.de (A.K.); 2 Department for Womens Health, University Hospital Tübingen, 72076 Tübingen, Germany

**Keywords:** psychological stress, pregnancy, depression, midwifery, mental health, women*’s health

## Abstract

Background/Objectives: Prenatal psychology studies show that stress, depression, and psychological stress during pregnancy can have a significant impact on maternal and fetal health and are highly prevalent. The aim of the study was to compare maternal and neonatal short-term outcomes in pregnant women* (the asterisk (*) is used at the appropriate places in this text to indicate that all genders are included) with a history of prenatal maternal psychological stress (PMPS) with those of pregnant women* not exposed to PMPS to determine differences and identify risk factors. Methods: Statistical tests for differences and relative risks between the groups were carried out with the perinatal data of University Hospital Tübingen from 2022 using IBM SPSS. Results: The study shows that PMPS has significant negative effects on various parameters, including the rate of premature births, preeclampsia, induction of birth, birth duration, and fetal asphyxia, as well as the birth weight of the children and their Apgar values (an assessment of newborn health scored shortly after birth). In addition, the risk of PMPS increases in women* with stillbirths and two or more previous miscarriages. However, the practical relevance must be critically scrutinized and confirmed by bigger studies. Conclusions: PMPS has a significant impact on the maternal and neonatal birth outcomes and must be identified as a risk factor in pregnancy. There is still a need for further research with larger samples, more balanced groups, and multivariate regression models to generate detailed, more transferable results and a deeper insight into the significant effects of PMPS and the role midwives can play in helping it.

## 1. Introduction

**Figure 1:**
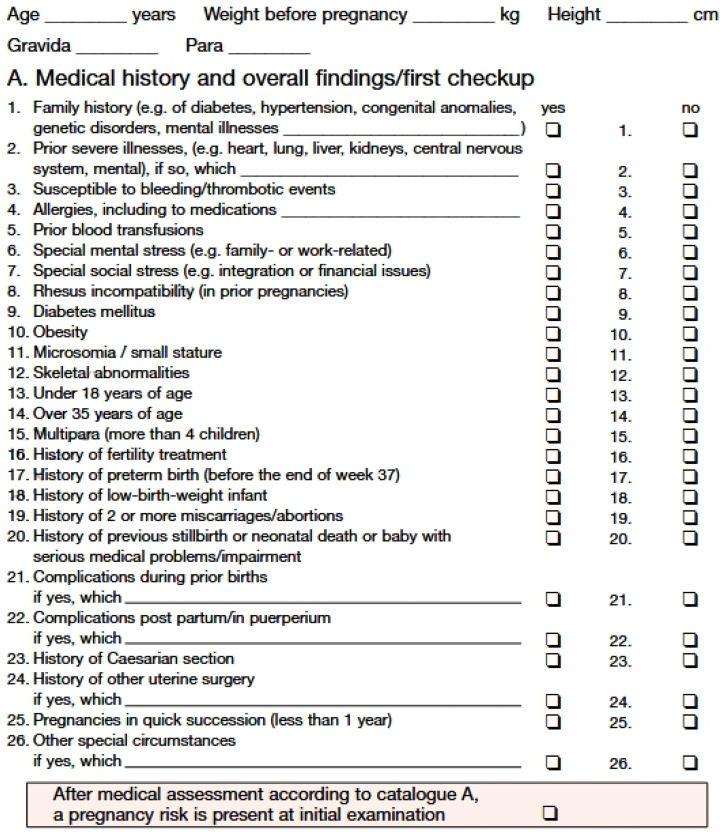
Risk catalogue A of the maternity record (Mutterpass) [3].

**Figure 2:**
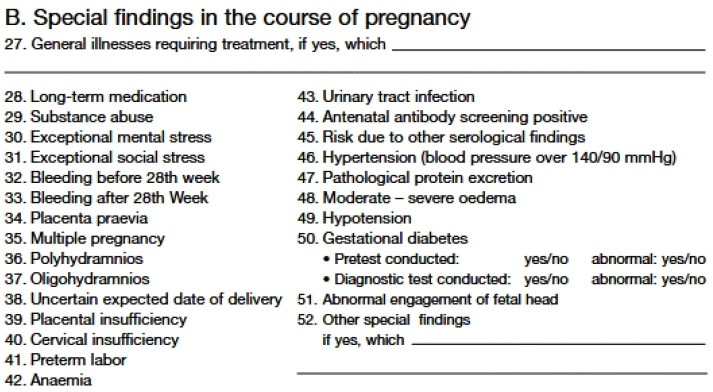
Risk catalogue B of the maternity record (Mutterpass) [3].

Studies on prenatal psychology show that stress, depression, and the woman*’s (the asterisk (*) is used at the appropriate places in this text to indicate that all genders are included) emotional state during pregnancy can have a significant impact on the condition of the fetus [1,2]. Both acute and chronic stress during pregnancy can cause an allostatic overload or a long-term imbalance in the mediators of homeostasis, leading to disturbances in maternal–placental–fetal endocrine and immunological responses [2]. The definition of psychological stress and what can be perceived as such is very complex and depends on numerous factors, which is why there is no standard definition and classification. In this study it is defined as all aspects and situations that are listed in risk catalogues A and B of the maternity passport under points 6, 7, 30, and 31, which include “special mental and social stress” and “exceptional mental and social stress”, as in Germany, a maternity record known as “Mutterpass” is kept for every pregnant person [3]. The maternal anamnesis documented in the “Mutterpass” can be seen in Figure 1 and Figure 2.

These risk points explicitly include medically diagnosed mental illnesses, such as depression, posttraumatic stress disorders, and various anxiety disorders, but they are not described in more detail [4]. All these ailments, including an increased level of psychological stress perceived by pregnant people without a psychiatric diagnosis, are summarized under the term prenatal maternal psychological stress (PMPS) in this study. As mentioned, in Germany, all medical diagnoses and relevant findings relating to pregnancy are documented exclusively in the “Mutterpass”. The definition of PMPS can therefore only be based on the risk factors recorded in the maternity record (Mutterpass) and as can be seen in Figure 1 and Figure 2, a cross is only marked if there is psychological or social stress, but the exact cause of PMPS is not documented and therefore cannot be used for research purposes. As a result, the definition of PMPS is not an officially recognized diagnostic term within major psychiatric and medical classification systems such as the DSM-5, ICD-10, or ICD-11. Rather, it is a research construct that is not clinically standardized. This lack of uniformity can lead to the term PMPS being interpreted and applied differently in different studies, which must be taken into account when interpreting the present study.

The reasons for PMPS are complex and multifactorial; it can be caused by complicated family situations, partnership conflicts, single parenting, domestic or sexualized abuse, trauma, refugee experience, traumatic birth, financial difficulties, fertility problems, and many other circumstances such as sexism and racism [1,5,6,7,8,9]. These influencing factors are comparable to the social determinants of health described by the World Health Organization and have a huge impact on the health condition and health behavior of the people concerned [10].

Women* who have already given birth to a stillborn or impaired child and those who have a history of miscarriages are considered to be particularly at risk of psychological stress in a subsequent pregnancy as a result of their experience and often traumatization [1,7,11]. Pregnant women* who had to undergo fertility treatment to become pregnant also belong to this group as they often experience depression and anxiety symptoms as a result of the hormonal stimulation [6]. Sexualized and physical violence against women* must also be seen as a major risk factor for PMPS and remains ever-present in today’s society [12]. Thirty-three percent of women* living in Germany have been victims of physical and/or sexual violence by the age of 15 [6]. Five percent of women* aged 15 and over have been raped and fifty-five percent of women* state that they have experienced sexual harassment [6]. Forty-three percent of all women* in Germany who have experienced violence state that it was perpetrated by their (ex-)partner and often in a combination of sexual and physical violence [6]. The issue of violence is also omnipresent during pregnancy, with a prevalence of around 35% in Muslim countries and 6% in Europe, in some cases even higher than that of non-pregnant women* [13,14]. Factors that are associated with a higher risk of abuse during pregnancy are nationality, living in urban areas, a low socio-economic background, and a low level of education [13,15]. Women* with a migrant background or with a partner with a migrant background, unemployed people, homemakers, and students also have a higher risk of being exposed to violence during pregnancy as well as women* who have a significant age difference (≥10 years) between them and their partner, a history of abortions, and an unwanted pregnancy [15]. Women* who suffer from domestic and sexual abuse are significantly more likely to develop symptoms of depression and anxiety, also in pregnancy [16].

It must be considered that pregnant women* may be reluctant to share symptoms of sadness and irritability, as depression is associated with shame due to stigma [7]. There is also a discrepancy between the expected happy feelings during pregnancy and women’s* actual experiences [17]. The role model of the “good mother”, which is characterized by numerous conservative, ecclesiastical, patriarchal, and also feminist demands, puts many mothers* under enormous pressure, as they want to conform to this expression in order to avoid social disapproval or social ostracism as well as to receive recognition and appreciation for their work [18,19,20]. Nevertheless, this appreciation does not materialize, as the image of the perfect mother* seems unattainable and is inherently contradictory [17,20]. They are supposed to be a career woman, homemaker and mother* at the same time, all without neglecting any part of their life and bereft of complaint [17,20]. It is demanded that with the onset of pregnancy, the person suppresses all needs for autonomy, self-realization, intellectual activity, and financial reward and gives everything to satisfy and fulfil this paradoxical social demand, as being a good mother seems incompatible with desiring time alone and seeking help from others [20,21].

In addition, there is a tendency to focus on physical (maternal and fetal) health during pregnancy rather than mental health and to incorrectly attribute emotional complaints to physical and hormonal changes during pregnancy [7,21]. In fact, pregnant women* often show atypical symptoms of depression and unspecified somatic complaints such as fatigue, loss of energy, appetite and sleep disturbances, and depressed mood [7,22]. Pregnancy anxiety, characterized by the anticipation of future threats related to childbirth and parenting, often arises from fears of complications, disability in the child, and pregnancy-related changes [23]. Although anxiety can serve as a coping mechanism for expectant mothers*, it may evolve into a disorder, impacting mental health and leading to negative birth outcomes [23,24]. Various factors contribute to this anxiety, including fear of pain, past traumatic (birth) experiences, personality traits, psychosocial issues, perceived lack of support by the social environment, low education levels, and exposure to alarming birth stories from other people. The frequency and intensity of anxiety are influenced by each woman*’s perception of stressors and her coping abilities [23].

It can therefore be difficult to distinguish between physiological pregnancy symptoms, which often occur, and atypical somatic complaints, which may be related to depression or anxiety [7,24,25]. In pregnancy, the prevalence of depression is estimated to be between 7% and 20% in high-income countries [2,7,21,24,26], while rates of 25% or more have been reported in low- and middle-income countries [7,21]. Approximately 10% of pregnant women* meet the criteria for major depression [2], and in 50% of women* prenatal depression persists and continues into the postnatal period [21]. Prenatal exposure to adverse environmental conditions can also affect fetal development via physiological intrauterine mechanisms, while the postnatal environment can affect child development through negative parental behaviors and lack of care [27]. In addition to direct biological effects of maternal stress on fetal and infant neurodevelopment, numerous environmental and socio-economic factors also interact with prenatal stress, depression, or anxiety and can increase this influence [27]. The environment can either serve as a buffer for maternal stress or reinforce its effects on child development [27]. PMPS is associated with a significantly lower birth weight of the child, an increased rate of preeclampsia, inductions and c-sections, more miscarriages and premature births, as well as more complicated births with more interventions needed [2,14,28,29,30,31]. Furthermore, an increase in neonatal morbidity (as evidenced by a higher incidence of asphyxia and lower Apgar scores), a lower breastfeeding rate, reduced milk production, and mother–infant bonding problems have been linked to PMPS [2,32,33,34]. The aim of this retrospective study is to differentiate the impact that PMPS has on birth outcomes and to increase awareness, especially among midwives, to impart knowledge about the risk groups and the consequences, and to identify further research needs for midwifery science. Only if midwives meet these requirements can their function as gatekeepers be fulfilled.

## 2. Materials and Methods

### 2.1. Study Design

This retrospective cohort study was conducted in 2024 and analyzed perinatal data from the University Hospital of Tübingen, Baden-Württemberg, Germany in 2022 to compare women* with perinatal psychological stress (PMPS) with women* without psychological stress during pregnancy.

### 2.2. Participants

The study population included all women* who were cared for in the maternity ward in 2022 at the University Hospital in Tübingen, Baden-Württemberg, Germany. The inclusion and exclusion criteria were based on the presence or absence of increased maternal psychological stress during pregnancy, which was determined on the basis of the entries in the maternity record (Mutterpass). Particularly, the sixth item (special psychological stress, e.g., family or occupational) and the seventh item (special social stress, integration problems, economic problems) under “General Findings”, as well as item 30 (psychological stress) and item 31 (special social stress) under “Special findings in the course of pregnancy”. Missing values were excluded from the analyses to avoid bias.

### 2.3. Instruments

These items were used to recode the general variable “PMPS” using Microsoft Excel (version 16.89.1), Microsoft corporation, Redmond, WA, USA, which included all women* who, according to the risk catalogue in their maternity passports (Mutterpass), were affected by one or more of the items mentioned. The variable PMPS is not based on ICD codes because the data set did not include them and, in Germany, the pregnancy-related medical anamnesis is kept in the “Mutterpass”, which does not include ICD codes for psychological conditions during pregnancy [3].

Similarly, other necessary variables in the data set were recoded using admission, birth, and discharge diagnosis keys to be able to perform calculations with them. If there were missing values in the variables, these were excluded from the calculations and the fields were left blank so as not to distort the results. At the time of the start of the calculations, an approved ethics application from the Ethics Committee of the University Hospital of Tübingen was available, which authorized the retrospective processing of the data for this study design and the answering of the research question. After approval by the ethics committee, the required data parameters were read anonymously from the data sets routinely collected as part of quality assurance. They were stored locally on a password-protected computer with a server in Germany, to which only the researchers had access. They were used for the duration of the evaluation and processing time of the study and then irrevocably deleted. The statistical analysis was carried out using Microsoft Excel version 16.89.1, Microsoft corporation, Redmond, WA, USA, and IBM SPSS (version 29.0.2.0, IBM, Armonk, NY, USA,) and a significance level of 5% and a confidence interval of 95% were defined in advance.

### 2.4. Data Analysis

At the beginning, hypotheses regarding differences and correlations in the groups were formed for all variables. These can be investigated in Table 1 including the performed statistical tests and the data type of the variables. Chi-square tests were used for categorical variables, while unpaired *t*-tests or Mann–Whitney U tests were used for numerical variables, depending on the distribution [35]. The *p*-value indicates whether the differences found between the groups are statistically significant or not [36]. If the group difference was significant, the relative risk was calculated for categorical variables, as this has the best informative value in cohort studies and corresponds best to the study design [36]. In addition, the absolute and relative numbers of incidences in the groups were calculated and presented.

## 3. Results

**Figure 3:**
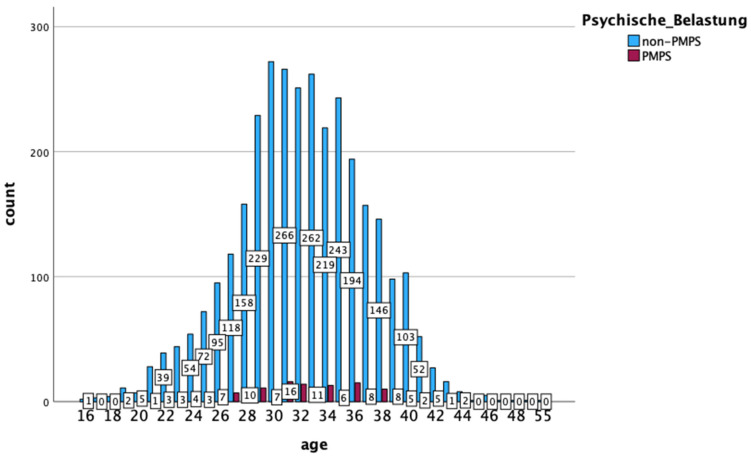
Distribution of maternal age (IBM SPSS).

The mothers* included in this study (n = 3368) were between 16 and 55 years old, the average age was 32. Most of the women* were aged between 29 and 36, and their due dates ranged from 15 December 2021 to 1 May 2023. The sex of all participants was stated as female, the gender was not documented. In the entire sample, 180 women* (5.3%) reported psychological stress in their medical history or during pregnancy, while the remaining 3188 women* (94.7%) had none of these risks listed in their maternity record (Mutterpass). These numbers are already much lower than the expected 7–20% found in the literature for high-income countries such as Germany [7,23,26]. The women with PMPS were aged between 16 and 44, while most of them were between 28 and 34 years old. The distribution of the age of the mothers* in the sample is shown graphically in Figure 3.

### 3.1. Maternal Influential Factors for PMPS

The group comparison regarding two or more miscarriages (n = 208 out of total n = 3368) in the past showed a highly significant result in the chi-square test with *p* < 0.001. Of the pregnant women* with PMPS, 13.9% had two or more miscarriages in their medical history; of those without, the figure was significantly lower at 5.7%. Accordingly, women* with two or more miscarriages in their medical history have a 2.44 times increased risk of high psychological stress. The calculations with the variable dead/impaired child in the medical history yielded similar results (n = 102 out of total n = 3368). In the group of pregnant women* with PMPS, 7.2% had reported a dead or damaged child in their medical history. In the group of pregnant women* with no psychological stress, the figure was 2.8%, which was significantly lower. Affected pregnant women* in this sample have a significantly higher risk of psychological stress by a factor of 2.57 than those without such a case in their medical history. Regarding fertility treatment (n = 188 out of total n = 3368), 7.8% of the pregnant women* with psychological stress underwent such treatment. In the group of women* without PMPS, the figure was slightly lower at 5.5%. With *p* = 0.187, the difference in the groups did not yield a significant result. The results of the performed statistical tests can be seen in Table 2.

### 3.2. Maternal Outcomes of PMPS

The group comparison in relation to preeclampsia (n = 82 out of total n = 3368) shows that, of the pregnant women* with PMPS, 7.8% developed preeclampsia, while only 2.1% of those without PMPS were affected. The calculations show that PMPS has a significant influence on the development of preeclampsia and the women* affected have a 3.71 times higher risk compared to women* without PMPS, which coincides with the research. As expected, the calculations of the variables “PMPS” and “induction of birth” (n = 236 out of total n = 3324) showed significant results. Here, 11.7% of pregnant women* with PMPS were induced, compared to 6.8% of those without psychological stress. In this sample, PMPS had a significant influence on whether the birth was induced. The rate of induction was increased by 72% for pregnant women* with psychological stress. There were no significant differences in the groups regarding the mode of delivery and postpartum complications. Contrary to expectations, PMPS did not have an impact on the c-section rate in this study and did not affect the duration of the birth in a negative way. In fact, the women* with PMPS were in labor for a shorter amount of time, with an average duration of 6.67 h compared to 8.36 h in the other group. The data gained in the calculations are shown in Table 3.

### 3.3. Neonatal Outcomes of PMPS

In the group of women* with PMPS, the rate of premature births (n = 378 out of total n = 3368) was significantly higher at 27.8% than in the group not affected by PMPS at 10.3%. Judging by the results of the regression, women* with PMPS appear to have a 2.6-fold higher risk of suffering a premature birth (<37 weeks) than those without. In addition to the low explanatory power, it must be noted in the interpretation that in this study there was no division into very preterm (<28 + 0 weeks’ gestation), early preterm (>28 + 0 weeks’ gestation <34 + 0 weeks’ gestation), and late preterm (>34 + 0 weeks’ gestation <37 + 0 weeks’ gestation) infants [37] whereby the outcome and prognosis, as well as mortality, can strongly depend on when exactly the child was born [38]. As expected, the children in the cohort of psychologically stressed women* were significantly lighter on average than the children of mothers* without PMPS (PMPS MID = 3100 g vs. non-PMPS MID = 3235 g). The birth weights were not divided into their percentiles, so it is not possible to say if there were significantly more small for gestational age (SGA) children in the PMPS group. In the group of women* with PMPS, 5.1% gave birth to a child who suffered asphyxia (n = 11 out of total n = 3277) at birth while, in the group of women* without, it was 0.1%. According to this, pregnant women* with PMPS have a 51-fold increased risk of giving birth to an asphyxiated child than those not affected. The differences in the group sizes dampen the significance of the *p*-value and the high RR must be interpreted carefully as the relevance for practice may be low. Apgar scores (an assessment of newborn health scored shortly after birth) showed significantly lower values in children of mothers with PMPS at both 1 min (PMPS 7.7 vs. non-PMPS 8.4; *p* = 0.002) and 10 min (PMPS 9.2 vs. non-PMPS 9.7; *p* = 0.004) compared to the children of unaffected mothers*. There does not appear to be a significant difference in the 5-min Apgar scores (PMPS 9.1 vs. non-PMPS 9.3; *p* = 0.28. The calculation results are shown in Table 4.

## 4. Discussion

The effects of PMPS on birth outcomes could be determined based on the statistical analyses that showed that women* with PMPS were significantly more likely to suffer from preeclampsia, required induction of labor more frequently, and more often had a damaged or deceased child or two or more miscarriages in their medical history. In addition, the relative risk of premature births was significantly higher in this group. The newborns of these women* had a significantly lower birth weight and lower 1- and 10-min Apgar values and were more likely to suffer asphyxia during birth.

Contrary to expectations, the sample showed that the duration of birth was slightly shorter in women* with PMPS. However, no significant differences were found between psychologically stressed and non-stressed women* in terms of the mode of delivery, postpartum complications and 5-min Apgar scores.

### 4.1. Maternal Influential Factors

First, the risk factors that can promote the occurrence of PMPS are discussed. In this study, it was found to be very complicated and difficult to filter out and categorize the women* with mental illnesses such as depression, PTSD, anxiety, and panic disorders individually, as the medical history was not filled out in sufficient detail or this was not transferred to the birth documentation program in sufficient detail. The data set showed that some mental illnesses were listed under item 2 of risk catalogue A “previous own illnesses”, but the majority were not, which made it difficult to classify the PMPS. For this reason, those risk factors of PMPS described in the literature that are listed separately in the risk catalogues of the maternal passport were extracted. These are “history of 2 or more miscarriages”, “history of stillborn or dead/impaired child”, and “fertility treatment” [2,25,26]. It is estimated that around 20% of clinically recognized pregnancies and up to half of all pregnancies end in miscarriage [38]. This experience can have a significant impact on the psychological well-being of the women* affected and is often associated with increased stress, anxiety, and depression, which in some cases can last up to a year. In addition, it is increasingly recognized that the psychological consequences of a miscarriage can persist in subsequent pregnancies and lead to considerable stress [38]. In Germany, however, a miscarriage is only recorded as a risk factor in the maternity record if there is a history of two or more miscarriages [3]. The study confirms that women* with two or more miscarriages have a 2.4-fold increased risk of psychological stress during pregnancy (PMPS 13.9% vs. non-PMPS 5.7%; *p* < 0.001, RR 2.44) which is consistent with the literature, as there is evidence that risk for PMPS increases after one miscarriage [39]. Stillbirth, although comparatively common, is often considered less significant than other types of child loss [40]. However, studies show that mothers* who have experienced a stillbirth often suffer from anxiety and depressive symptoms for up to three years [11,40]. They are also at risk of suffering from anxiety and posttraumatic stress disorder during a subsequent pregnancy or after the birth of a healthy child [11,40]. According to this study, women* with a conspicuous history of stillbirth or a damaged child also have a significantly increased risk (PMPS 7.2% vs. non-PMPS 2.8%; *p* < 0.001; RR 2.57) of PMPS in subsequent pregnancies.

Furthermore, it is often described that women* who undergo fertility treatment suffer from increased psychological stress during pregnancy [41]. This is attributed to increased depression and anxiety as well as increased physical complaints that occur as a result of the diagnostic and therapeutic measures, particularly hormonal stimulation [6]. However, the results of the present study contradict these assumptions. Although a difference was found between the groups (PMPS: 7.8% vs. no PMPS: 5.5%), and proportionally more women* who had undergone fertility treatment were psychologically stressed during pregnancy, this difference was not statistically significant (*p* = 0.19). Furthermore, it should be critically viewed that due to a lack of information in the maternity record and insufficient information from the risk catalogues, no differentiation could be made regarding the type of infertility treatment performed and whether hormonal stimulation was required, which might have biased the results.

It should also be noted that the socio-demographic characteristics like gender, ethnicity, socio-economic status, and age were not taken into account in this study, which makes the results less accurate as, for example, studies show that race-related stress contributes to psychological stress and physical illness in African American individuals [9]. Previous research links vigilance and hypervigilance in African American women* to depression and increased psychological distress, emphasizing how relevant social stressors are in the context of ethnicity and gender [9]. Furthermore, the population of the Tübingen district, and consequently the clientele of the women*’s clinic delivery ward, is predominantly composed of white, affluent academics, rendering it an inadequate representation of the German population as a whole [42].

In addition, the definition of PMPS is not an officially recognized diagnostic term within major psychiatric and medical classification systems. Rather, it is a research construct that is not clinically standardized. This lack of uniformity can result in the term PMPS being interpreted and applied differently in different studies, which must be taken into account when interpreting the results of the present study.

### 4.2. Maternal and Neonatal Outcomes

The development of preeclampsia is a major risk factor of PMPS, as chronic stress, especially in early pregnancy, is associated with a significantly increased rate of preeclampsia and increases the risk by up to 62% [31,43]. The results of this study therefore affirm previous scientific findings regarding PMPS and preeclampsia, as the risk of developing preeclampsia was 3.71-fold higher in the group of women* with PMPS (PMPS 7.8% vs. non-PMPS 2.1%; *p* < 0.001; RR 3.71). However, it should be noted that the model has a low explanatory power in this study, as can be seen from the differences in the group sizes. There is a need for research into the causes of this correlation and which risk factors could additionally influence it. Further risks associated with the development of preeclampsia, but also with PMPS, are premature birth and a low birth weight [2]. In a large cohort of women* screened for depression before birth, women* with depressive symptoms were more likely to have a (very early) preterm birth and an SGA newborn [44]. Generalized anxiety disorder during pregnancy appears to increase the likelihood of delivering a low birth weight or small for gestational age infant, while PTSD is associated with an increased likelihood of preterm birth [28]. The results of this study are congruent with the literature (PMPS MID = 3100 g vs. non-PMPS MID = 3235 g; *p* = 0.01) but as the birth weights were not divided into their percentiles, it is not possible to say which of the children were SGA children. Therefore, there is a big need for research into which types of psychological stress may have a particular influence on the birth weight factor, how strong this negative correlation is in larger samples, and whether significantly more children become SGA children as a result as there is evidence that, after adjustment, the women* who experience high levels of PMPS have an increased risk of delivering a child with macrosomia [45]. Furthermore, the literature describes PMPS as a risk factor for premature birth [2,36], especially if the pregnant woman* is single or receives no support from her social environment or in her partnership [36]. This fact is reflected in the research results of this study. The women* with psychological stress had a 2.6-fold higher risk of suffering a premature birth than those in the cohort without PMPS (PMPS 27.8% vs. non-PMPS 10.3%; *p* = 0.01; RR 2.6). Regarding the points just mentioned and the results of the study conducted here, there is a big need for further research with larger samples and multivariate regression models to generate detailed and transferable results and a deeper insight into the significant effects of PMPS that are implied in this study. To determine whether this effect can be attributed to the impact of PMPS, multivariate regression analyses with variables such as preeclampsia, birth weight, and preterm birth are necessary to rule out confounding factors

There is evidence that obstetric interventions are more frequent in the births of pregnant women* with PMPS and that they are more often necessary than in the births of other mothers* [29]. This also includes the induction of labor with medication, an increased need for c-sections and vacuum extractions, the duration of the birth, and postpartum complications [29]. The data generated in this study were not congruent to the findings in the literature as there were no significant differences regarding the mode of delivery and postpartum complications and women* with PMPS had a significantly shorter duration of labor (PMPS MID = 6.7 h vs. non-PMPS MID = 8.4 h; *p* = 0.002). Women* with PMPS were induced significantly more in this sample (PMPS 11.7% vs. non-PMPS 6.8%; *p* = 0.014; RR 1.72), but the model has very low explanatory power and the relevance for practice must also be considered low due to the differences in the group sizes and the fact that the medical reasons why the obstetric team indicated the induction are not clear from the data set and are multifactorial. As pregnancy appears to be more difficult overall for women* with PMPS, it is reasonable to assume that they may wish to end it earlier at their own request [46]. Another impact of PMPS is that on neonatal morbidity as evidenced by lower Apgar scores and an increased rate of asphyxia among those affected [2,47]. The study shows that children of psychologically stressed mothers* may take longer to adjust after birth, as both the 1-min Apgar scores (PMPS 7.7 vs. non-PMPS 8.4; *p* = 0.002) and the 10-min Apgar scores (PMPS 9.2 vs. non-PMPS 9.7; *p* = 0.004) were significantly lower, while no significant difference was found in the 5-min scores [2]. This may suggest that children of psychologically stressed mothers* more often experience adjustment disorder after birth and must endure more complicated births, whereas the effect size and thus the practical relevance can be considered low [2].

In addition, a significantly higher incidence of asphyxia was observed in psychologically stressed mothers (PMPS 5.1% vs. non-PMPS stress 0.1%; *p* < 0.001; RR 51), which shows a significantly increased risk of fetal asphyxia at birth, although the difference in size of the groups may distort the results and relativizes the effect size, so further studies with similar group sizes are necessary to validate these results.

### 4.3. Limitations and Strengths

PMPS encompasses a broad spectrum of stressors, ranging from mental health conditions such as anxiety and depression to social stress, trauma, and financial difficulties. This broad definition can dilute the specificity of the concept, making it difficult to establish precise assessment criteria and problematic to consider the specific impact of PMPS on pregnancy outcomes in isolation. PMPS is often assessed using variable and sometimes subjective measures of stress or distress without standardized diagnostic tools, which can lead to inconsistencies in research results and their interpretation. In addition, there are established terms in psychiatry and obstetrics for mental illnesses during pregnancy, such as perinatal depression and anxiety disorders. However, due to the documentation methods used in Germany, these could not be extracted individually from the data set and therefore not be used for calculations. PMPS may overlap with these diagnoses, but it lacks specific diagnostic criteria, making it difficult and potentially ambiguous to use in a clinical context. This must be seen as a great limitation of this study, as the definition of women* with PMPS was based solely on the medical history in the maternity record (Mutterpass). The risk catalogue in the maternity record has not been revised or adapted since 1980, which is why its practical application and the resulting findings should generally be viewed critically [3]. There are also indications that the medical history taken by midwives and gynecologists during pregnancy is inadequate and that important aspects, such as the occurrence of mental illness in the family or drug or alcohol consumption, are not asked about and documented [6]. Therefore, it cannot be assumed that the number of mentally stressed women* recorded in this study is representative of the population. In the literature, 7–20% of pregnant women* are described as having a particular psychological burden or signs of depression during pregnancy, which is in big contrast to the 5.3% of pregnant women* affected here [7,23]. Furthermore, it must be criticized that the research in literature, which forms the basis of this work, was not systematic and therefore cannot be reproduced comparatively. The study benefited from the approval of the ethics committee for the handling of sensitive health data. Despite a relatively large sample, the proportion of women experiencing psychological stress was lower than expected. Nonetheless, the results provide valuable insights into the effects of psychological stress during pregnancy on maternal and child health and are generally consistent with the existing literature. One limitation, however, is the different group sizes (PMPS 180 vs. non-PMPS 3183), as these could affect the statistical significance of the observed differences. To further validate these results, additional analyses with larger and more balanced groups and multivariate regression models including social and socio-economic factors of the individuals concerned as well as the other variables like induction of birth, preeclampsia, preterm birth, and so on and are required.

## 5. Conclusions

The research question can be answered in the affirmative, as the present study shows that PMPS has significant negative effects on various parameters, including the rate of premature births, preeclampsia, induction of birth, birth duration, and fetal asphyxia, as well as the birth weight of the children and their Apgar values. In addition, the risk of PMPS increases in women* with stillbirths and two or more previous miscarriages. The present findings emphasize the need for further research with larger samples and multivariate regression models to obtain detailed and transferable results and to gain a better understanding of the significant impact of PMPS. There is ample evidence of the critical importance of midwives’ contribution to achieving better health outcomes for women*, newborns, and their families [48]. It is therefore reasonable to postulate that without the contribution of midwives, who provide high-quality care, regarding PMPS, achieving the global health agenda for women* and newborns seems unattainable [48].

## Figures and Tables

**Table 1:** Variables, hypothesis, and performed statistical analysis.

Variable	Hypothesis 1: Differences	Hypothesis 1: Correlation	Data Type/Information		Statistical Tests
Prenatal maternal psychological stress (PMPS)	-	-	Categorical, nominal		-
Preeclampsia	There is a significant difference between the groups with and without PMPS regarding the occurrence of preeclampsia.	There is a significant correlation between the occurrence of PMPS and preeclampsia.	Categorical	Nominal	Chi-square test, RR
Induction of birth	There is a significant difference between the groups with and without PMPS regarding the induction rate.	There is a significant correlation between the occurrence of PMPS and induction of birth.	Categorical	Nominal	Chi-square test, RR
History of two or more miscarriages	There is a significant difference between the groups with and without PMPS regarding miscarriages in the anamnesis.	There is a significant correlation between the occurrence of PMPS and miscarriages in the anamnesis.	Categorical	Nominal	Chi-square test, RR
History of stillbirth or dead/impaired child	There is a significant difference between the groups with and without PMPS regarding a dead/impaired child in the anamnesis.	There is a significant correlation between the occurrence of PMPS and a dead/impaired child in the anamnesis.	Categorical	Nominal	Chi-square test, RR
Fertility treatment	There is a significant difference between the groups with and without PMPS regarding fertility treatments.	There is a significant correlation between the occurrence of PMPS and fertility treatment.	Categorical	Nominal	Chi-square test, RR
Preterm birth	There is a significant difference between the groups with and without PMPS regarding preterm birth.	There is a significant correlation between the occurrence of PMPS and preterm birth.	Categorical	Nominal	Chi-square test, RR
Birth weight	There is a significant difference between the groups with and without PMPS regarding the child′s birth weight.	There is a significant correlation between the occurrence of PMPS and the child′s birth weight.	Numeric	Continuo-us, non-parametric	Mann–Whitney U test
Asphyxia	There is a significant difference between the groups with and without PMPS regarding asphyxia.	There is a significant correlation between the occurrence of PMPS and asphyxia.	Categorical	Nominal	Chi-square test, RR
Apgar 1′	There is a significant difference between the groups with and without PMPS regarding the 1 min Apgar.	There is a significant correlation between the occurrence of PMPS and the 1 min Apgar.	Numeric	Discrete, non-parametric	Mann–Whitney U test
Apgar 5′	There is a significant difference between the groups with and without PMPS regarding the 5 min Apgar.	There is a significant correlation between the occurrence of PMPS and the 5 min Apgar.	Numeric	Discrete, non-parametric	Mann–Whitney U test
Apgar 10′	There is a significant difference between the groups with and without PMPS regarding the 10 min Apgar.	There is a significant correlation between the occurrence of PMPS and the 10 min Apgar.	Numeric	Discrete, non-parametric	Mann–Whitney U test
Duration of birth	There is a significant difference between the groups with and without PMPS regarding the duration of birth.	There is a significant correlation between the occurrence of PMPS and the duration of birth.	Numeric	Continuous, non-parametric	Mann–Whitney U test
Mode of delivery	There is a significant difference between the groups with and without PMPS regarding the mode of delivery.	There is a significant correlation between the occurrence of PMPS and the mode of delivery.	Categorical	Nominal	Chi-square test, RR
Postpartum complications	There is a significant difference between the groups with and without PMPS regarding the rate of postpartum complications.	There is a significant correlation between the occurrence of PMPS and the rate of postpartum complications.	Categorical	Nominal	Chi-square test, RR

**Table 2:** Maternal influential factors for PMPS.

Variable	n/%		Chi-Square Test	RR
	PMPS	Non-PMPS		
Two or more miscarriages in the anamnesis	25/13.9%	183/5.7%	*p* < 0.001	2.44
Dead/impaired child in the anamnesis	13/7.2%	89/2.8%	*p* < 0.001	2.57
Fertility treatment	14/7.8%	174/5.5%	*p* = 0.187	-

**Table 3:** Maternal outcome of PMPS.

Variable	n/%		Chi-Square Test	RR
	PMPS	Non-PMPS		
Preeclampsia	14/7.8%	68/2.1%	*p* < 0.001	3.71
Induction of birth	21/11.7%	215/6.8%	*p* = 0.014	1.72
Mode of delivery	Primary c-section: 21/11.7% Secondary c-section: 43/23.9% Vacuum-delivery: 32/17.8% Spontaneous delivery: 84/46.7%	Primary c-section: 448/13.3% Secondary c-section: 775/23% Vacuum-delivery: 593/17.6% Spontaneous delivery: 1546/45.9%	*p* = 0.907	-
Postpartum complications	1/0.6%	49/1.6%	*p* = 0.282	-
	-	-	**Mann–Whitney** **U test**	-
Duration of birth in hours	MID = 6.7 h (median)	MID = 8.4 h	*p* = 0.002	-

**Table 4:** Neonatal outcomes of PMPS.

Variable	n/%		Chi-Square Test	RR
	PMPS	non-PMPS		
Preterm birth	50/27.8%	328/10.3%	*p* = 0.01	2.6
Asphyxia	9/5.1%	2/0.1%	*p* < 0.001	51
			**Mann–Whitney** **U test**	
Birth weight in g	MID = 3100 g (median)	MID = 3235 g	*p* = 0.01	-
Apgar 1′ score	MID = 7.7	MID = 8.4	*p* = 0.002	-
Apgar 5′ score	MID = 9.1	MID = 9.3	*p* = 0.28	-
Apgar 10′ score	MID = 9.2	MID = 9.7	*p* = 0.004	-

## Data Availability

Data available on request due to ethical restrictions.

## References

[1] Bjelica A., Cetkovic N., Trninic-Pjevic A., Mladenovic-Segedi L. (2018). The phenomenon of pregnancy—A psychological view. Ginekol. Pol..

[2] Traylor C.S., Johnson J.D., Kimmel M.C., Manuck T.A. (2020). Effects of psychological stress on adverse pregnancy outcomes and nonpharmacologic approaches for reduction: An expert review. Am. J. Obstet. Gynecol. MFM.

[3] Gemeinsamer Bundesausschuss Mutterpass 2020. https://www.g-ba.de/downloads/83-691-594/2020-02-20_G-BA_Mutterpass_web.pdf.

[4] Fawcett E.J., Fairbrother N., Cox M.L., White I.R., Fawcett J.M. (2019). The Prevalence of Anxiety Disorders During Pregnancy and the Postpartum Period: A Multivariate Bayesian Meta-Analysis. J. Clin. Psychiatry.

[5] Tang X., Lu Z., Hu D., Zhong X. (2019). Influencing factors for prenatal Stress, anxiety and depression in early pregnancy among women in Chongqing, China. J. Affect. Disord..

[6] Robert-Koch-Institut (2020). Gesundheitsberichterstattung Des Bundes Gemeinsam Getragen Von Rki Und Destatis Gesundheitliche Lage der Frauen in Deutschland.

[7] Biaggi A., Conroy S., Pawlby S., Pariante C.M. (2016). Identifying the women at risk of antenatal anxiety and depression: A systematic review. J. Affect. Disord..

[8] Deutsche Gesellschaft Für Psychiatrie Und Psychotherapie, Psychosomatik Und Nervenheilkunde (DGPPN), Bundesärztekammer (BÄK), Kassenärztliche Bundesvereinigung (KBV), Arbeitsgemeinschaft Der Wissenschaftlichen Medizinischen Fachgesellschaften (AWMF), Ärztliches Zentrum Für Qualität In Der Medizin (ÄZQ) (2015). S3-Leitlinie/Nationale VersorgungsLeitlinie Unipolare Depression-Langfassung.

[9] Hunter E.A., Spears E.C., Martz C.D., Chung K., Fuller-Rowell T.E., Lim S.S., Drenkard C., Chae D.H. (2021). Racism-related stress and psychological distress: Black Women’s Experiences Living with Lupus study. J. Health Psychol..

[10] WHO (2008). Closing the Gap in a Generation: Health Equitiy Through Action on the Social Determinants of Health.

[11] Mergl R., Quaatz S.M., Lemke V., Allgaier A.-K. (2024). Prevalence of depression and depressive symptoms in women with previous miscarriages or stillbirths—A systematic review. J. Psychiatr. Res..

[12] Melville J.L., Gavin A., Guo Y., Fan M.-Y., Katon W.J. (2010). Depressive Disorders During Pregnancy: Prevalence and Risk Factors in a Large Urban Sample. Obstet. Gynecol..

[13] Habib S., Abbasi N., Khan B., Danish N., Nazir Q. (2018). Domestic Violence Among Pregnant Women. J. Ayub Med. Coll. Abbottabad JAMC.

[14] Arvanitidou O., Kosmas I., Michalopoulos C.-K., Doumanidou M., Ierodiakonou-Benou I., Athanasiadis A., Daniilidis A. (2023). The Impact of Stress and Depression on the Outcome of Human Gestation. Cureus.

[15] Antoniou E., Iatrakis G. (2019). Domestic Violence During Pregnancy in Greece. Int. J. Environ. Res. Public Health.

[16] Ghoneim H.M., Elprince M., Ali T.Y.M., Gharieb W.F., Ahmed A.A. (2021). Violence and depression among pregnant women in Egypt. BMC Pregnancy Childbirth.

[17] O’Brien K.M., Yoo S.-K., Kim Y.H., Cho Y., Salahuddin N.M. (2020). The Good Mothering Expectations Scale: An International Instrument Development Study. Couns. Psychol..

[18] Mundlos C. (2013). Mütterterror: Angst, Neid und Aggressionen Unter Müttern.

[19] Kahalon R., Preis H., Shilo G., Benyamini Y. (2021). Maternal Expectations Among Pregnant Women from Single, Lesbian, and Heterosexual Parented families. J. Fam. Issues.

[20] Henshaw E.J., Fried R., Teeters J.B., Siskind E.E. (2014). Maternal expectations and postpartum emotional adjustment in first-time mothers: Results of a questionnaire survey. J. Psychosom. Obstet. Gynecol..

[21] Rollè L., Giordano M., Santoniccolo F., Trombetta T. (2020). Prenatal Attachment and Perinatal Depression: A Systematic Review. Int. J. Environ. Res. Public Health.

[22] Posternak M.A., Zimmerman M. (2001). Symptoms of atypical depression. Psychiatry Res..

[23] Davoudi M., Pouladi Rishehri A., Danesh E. (2023). The Effectiveness of Narrative Exposure Therapy on Pregnancy Concerns, Schemas Coping with Stress and Interpersonal Sensitivity in Pregnant Women with Pregnancy Anxiety. Psychol. Woman J..

[24] Rondung E., Massoudi P., Nieminen K., Wickberg B., Peira N., Silverstein R., Moberg K., Lundqvist M., Grundberg Å., Hultcrantz M. (2024). Identification of depression and anxiety during pregnancy: A systematic review and meta-analysis of test accuracy. Acta Obstet. Gynecol. Scand..

[25] Marchesi C., Bertoni S., Maggini C. (2009). Major and Minor Depression in Pregnancy. Obstet. Gynecol..

[26] Andersson L., Sundström-Poromaa I., Wulff M., Åström M., Bixo M. (2006). Depression and anxiety during pregnancy and six months postpartum: A follow-up study. Acta Obstet. Gynecol. Scand..

[27] Bleker L.S., De Rooij S.R., Roseboom T.J. (2019). Prenatal Psychological Stress Exposure and Neurodevelopment and Health of Children. Int. J. Environ. Res. Public Health.

[28] Gelaye B., Sanchez S.E., Andrade A., Gómez O., Coker A.L., Dole N., Rondon M.B., Williams M.A. (2020). Association of antepartum depression, generalized anxiety, and posttraumatic stress disorder with infant birth weight and gestational age at delivery. J. Affect. Disord..

[29] Bayrampour H., Salmon C., Vinturache A., Tough S. (2015). Effect of depressive and anxiety symptoms during pregnancy on risk of obstetric interventions. J. Obstet. Gynaecol. Res..

[30] Hall W.A., Stoll K., Hutton E.K., Brown H. (2012). A prospective study of effects of psychological factors and sleep on obstetric interventions, mode of birth, and neonatal outcomes among low-risk British Columbian women. BMC Pregnancy Childbirth.

[31] Barrett E.S., Vitek W., Mbowe O., Thurston S.W., Legro R.S., Alvero R., Baker V., Bates G.W., Casson P., Coutifaris C. (2018). Allostatic load, a measure of chronic physiological stress, is associated with pregnancy outcomes, but not fertility, among women with unexplained infertility. Hum. Reprod..

[32] O’Dea G.A., Youssef G.J., Hagg L.J., Francis L.M., Spry E.A., Rossen L., Smith I., Teague S.J., Mansour K., Booth A. (2023). Associations between maternal psychological distress and mother-infant bonding: A systematic review and meta-analysis. Arch. Womens Ment. Health.

[33] Nagel E.M., Howland M.A., Pando C., Stang J., Mason S.M., Fields D.A., Demerath E.W. (2022). Maternal Psychological Distress and Lactation and Breastfeeding Outcomes: A Narrative Review. Clin. Ther..

[34] Döring N., Bortz J. (2016). Forschungsmethoden und Evaluation in den Sozial-und Humanwissenschaften.

[35] Häder M. (2019). Empirische Sozialforschung: Eine Einführung.

[36] Deutsche/Österreichische/Schweizerische Gesellschaft für Gynäkologie und Geburtshilfe S2 Leitlinie zur Prävention und Therapie der Frühgeburt 2022. https://register.awmf.org/assets/guidelines/015-025l_S2k_Praevention-Therapie-Fruehgeburt_2022-09.pdf.

[37] Yu Y., Cnattingius S., Olsen J., Parner E.T., Vestergaard M., Liew Z., Zhao N., Li J. (2017). Prenatal maternal bereavement and mortality in the first decades of life: A nationwide cohort study from Denmark and Sweden. Psychol. Med..

[38] San Lazaro Campillo I., Meaney S., McNamara K., O’Donoghue K. (2017). Psychological and support interventions to reduce levels of stress, anxiety or depression on women’s subsequent pregnancy with a history of miscarriage: An empty systematic review. BMJ Open.

[39] Farren J., Mitchell-Jones N., Verbakel J.Y., Timmerman D., Jalmbrant M., Bourne T. (2018). The psychological impact of early pregnancy loss. Hum. Reprod. Update.

[40] Horsch A., Jacobs I., McKenzie-McHarg K. (2015). Cognitive Predictors and Risk Factors of PTSD Following Stillbirth: A Short-Term Longitudinal Study. J. Trauma. Stress.

[41] AWMF (2019). S2k-Leitlinie Psychosomatisch Orientierte Diagnostik und Therapie bei Fertilitätsstörungen. https://register.awmf.org/de/leitlinien/detail/016-003.

[42] (2024). Statistischer Bericht Tübingen.

[43] Hux V.J., Roberts J.M. (2015). A Potential Role for Allostatic Load in Preeclampsia. Matern. Child Health J..

[44] Venkatesh K.K., Riley L., Castro V.M., Perlis R.H., Kaimal A.J. (2016). Association of Antenatal Depression Symptoms and Antidepressant Treatment With Preterm Birth. Obstet. Gynecol..

[45] Mélançon J., Bernard N., Forest J.-C., Tessier R., Tarabulsy G.M., Bouvier D., Giguère Y. (2020). Impact of maternal prenatal psychological stress on birth weight. Health Psychol..

[46] Pearlstein T. (2015). Depression during Pregnancy. Best Pract. Res. Clin. Obstet. Gynaecol..

[47] Lautarescu A., Craig M.C., Glover V. (2020). Prenatal stress: Effects on fetal and child brain development. International Review of Neurobiology.

[48] Kemp J., Maclean G.D., Moyo N. (2021). The Contribution of Midwifery to Global Health and Development. Global Midwifery: Principles, Policy and Practice.

